# Comparative Variation and Associations Among Seminal Microbiota, Oxidative Status, and Semen Quality in Different Rooster Types

**DOI:** 10.3390/ani16091380

**Published:** 2026-04-30

**Authors:** Supakorn Authaida, Wuttigrai Boonkum, Monchai Duangjinda, Vibuntita Chankitisakul

**Affiliations:** Department of Animal Science, Faculty of Agriculture, Khon Kaen University, Khon Kaen 40002, Thailand; supakorn.u@kkumail.com (S.A.); wuttbo@kku.ac.th (W.B.); monchai@kku.ac.th (M.D.)

**Keywords:** bacterial load, lipid peroxidation, nanopore sequencing, native chickens

## Abstract

This study examined the relationships among seminal bacterial composition, oxidative status, and semen quality in Thai native, crossbred, and commercial roosters. Fresh ejaculates (*n* = 10 per group) were evaluated for semen quality parameters—mass motility, total motility, progressive motility, sperm concentration, and viability and for malondialdehyde (MDA) levels, a marker of lipid peroxidation, as well as total bacterial load. Bacterial profiles were characterized using long-read 16S rRNA gene sequencing. Compared with commercial roosters, Thai native roosters exhibited significantly higher sperm motility and viability, along with markedly lower MDA concentrations (*p* < 0.05). Crossbred roosters showed intermediate values for most semen traits and oxidative indicators. Bacterial profiling revealed clear differences among rooster types in bacterial composition: commercial and crossbred roosters had higher bacterial richness and a greater prevalence of Gram-negative taxa, whereas Thai native roosters harbored lower bacterial diversity and load. Correlation analyses demonstrated that bacterial load was positively correlated with MDA concentration and negatively correlated with sperm motility and viability. These findings suggest that variation in seminal bacterial profiles, oxidative status, and semen quality is associated with differences among rooster types. The lower bacterial burden and lipid peroxidation observed in Thai native roosters may reflect greater reproductive resilience under tropical conditions, with potential implications for breeding and semen management strategies.

## Simple Summary

Semen quality is an important factor influencing reproductive success in poultry, and bacterial contamination is known to negatively affect sperm function. In birds, semen is particularly prone to microbial exposure because the digestive and reproductive tracts share a common opening. In this study, we investigated the relationships between seminal bacterial communities, oxidative status, and semen quality in three types of roosters raised under typical tropical production conditions. Differences in semen quality, bacterial profiles, and oxidative stress levels were observed among the groups. In general, higher bacterial load was associated with increased oxidative stress and reduced sperm motility and viability. However, because the birds were managed under observational conditions, the results should be interpreted as associations rather than direct causal effects of rooster type. These findings provide insight into how microbial contamination may be linked to semen quality in poultry and highlight the importance of considering both biological and management factors in semen handling and breeding practices.

## 1. Introduction

Bacterial contamination significantly affects semen quality and fertility in poultry. Since the gastrointestinal and reproductive tracts of birds converge at the cloaca, avian semen is inherently exposed to microbial contamination. This risk is amplified during semen collection and storage, as bacteria can proliferate in the nutrient-rich extenders commonly used for artificial insemination [1,2]. High bacterial loads impair sperm membrane integrity, reduce motility, and diminish fertilization potential by producing toxic metabolites and inducing oxidative stress [3,4].

Therefore, recent studies have focused on the composition and diversity of bacterial communities in poultry semen and their effects on semen quality and fertility [5]. For example, reports indicate that Lohmann Brown roosters harbor 27 bacterial species, ROSS 308 broiler breeders carry 16 species, and turkey semen contains 17 taxa [1,5]. Importantly, several of these taxa are associated with impaired sperm function.

However, most prior investigations have relied predominantly on culture-based approaches, which detect only viable and culturable bacteria and may overlook fastidious or non-culturable taxa. In addition, most studies have focused on a single breed or line, thereby limiting comparative insights across rooster types. To date, no studies have systematically characterized and compared the seminal bacterial profiles of Thai native, crossbred, and commercial roosters—three groups that differ not only in genetic background but also in broader physiological and production characteristics.

In contrast, long-read nanopore sequencing enables culture-independent characterization of microbial communities by directly sequencing full-length 16S rRNA genes, thereby detecting fastidious and non-culturable taxa that cannot be recovered using conventional culture-based methods. Furthermore, full-length sequencing improves taxonomic resolution, enabling more accurate species-level identification.

To address these gaps, we applied long-read nanopore sequencing of the 16S rRNA gene to profile bacterial DNA in rooster semen without culturing. We compared seminal bacterial composition across Thai native, crossbred, and commercial roosters, and examined associations among bacterial load, lipid peroxidation, and semen quality parameters. By integrating bacterial profiling with oxidative and reproductive assessments, this study characterized patterns among rooster types and explored relationships among bacterial load, oxidative status, and semen quality parameters.

## 2. Materials and Methods

### 2.1. Animals and Management

All experimental procedures were approved by the Institutional Animal Care and Use Committee of Khon Kaen University (IACUC-KKU-116/68).

Thirty roosters representing three rooster types were used: Thai native (Pradu Hang Dum), crossbred (Khai Mook Esarn KKU50; 50% Thai native blood), and commercial broiler-type (Arbor Acres), with 10 roosters per genotype. All roosters were 42 weeks of age at the time of sampling. All roosters were routinely managed for semen collection and had prior experience with the dorso-abdominal massage technique.

All birds were managed under standardized environmental and husbandry conditions at the Network Center for Animal Breeding and Omics Research (NCAB), Faculty of Agriculture, Khon Kaen University, Thailand. Roosters were individually housed in standardized cages (60 × 45 × 45 cm) within an open-sided housing system equipped with mechanical ventilation using exhaust fans to enhance air circulation and reduce heat accumulation. In addition, a roof-mounted evaporative cooling system (sprinkler system) was applied to further mitigate heat stress during high ambient temperatures. Environmental conditions were recorded during the one-month period preceding semen collection, the ambient temperature inside the house averaged 29.42 ± 2.13 °C, with relative humidity at 68.55 ± 3.56%, resulting in a calculated temperature–humidity index (THI) of 80.29 ± 3.85, calculated according to the National Oceanic and Atmospheric Administration [6]: THI = (1.8 × T + 32) − (0.55 − 0.0055 × RH) × (1.8 × T − 26), where T is ambient temperature (°C), and RH is relative humidity (%). Birds were maintained under natural lighting conditions, with an approximate photoperiod of 16 h light and 8 h dark.

All birds were fed a commercial breeder diet at a fixed feeding rate of 130 g per bird per day. The diet contained ≥17% crude protein, ≥3% crude fat, ≤8% crude fiber, and ≤12% moisture, with a metabolizable energy (ME) content of approximately 2800 kcal/kg, in accordance with the manufacturer’s specifications. The feeding regimen was strictly controlled and monitored daily to ensure consistent nutrient intake across all experimental units. Clean drinking water was provided ad libitum throughout the experimental period.

### 2.2. Experimental Design

A completely randomized design (CRD) was employed to compare semen quality, lipid peroxidation, and seminal bacterial profiles among three rooster genotypes, with individual roosters treated as independent experimental units.

One semen sample was collected from each rooster and evaluated for mass motility, total motility (MOT), progressive motility (PMOT), sperm concentration, and viability. The same ejaculates were further analyzed for lipid peroxidation, quantified as malondialdehyde (MDA) levels, and for total bacterial load using real-time quantitative PCR. Bacterial profiling was conducted using long-read 16S rRNA gene sequencing on the Oxford Nanopore platform (Oxford Nanopore Technologies (ONT), Oxford, UK).

Data on semen quality, bacterial load, lipid peroxidation, and bacterial diversity indices were integrated to explore associative relationships among three rooster types, bacterial burden, oxidative stress, and sperm functional traits. Heatmap visualization of bacterial detection frequencies was used to summarize prevalence patterns and support the ecological classification of bacterial taxa.

### 2.3. Semen Collection

Feathers surrounding the cloacal region were trimmed to reduce contamination, and the area was cleaned with sterile gauze to remove debris and fecal material. Semen was collected using the dorso-abdominal massage technique and immediately transferred into sterile 1.5 mL microcentrifuge tubes. Samples were kept at 22–25 °C and transported to the laboratory within 15 min. Upon arrival, semen samples were processed immediately, with aliquots allocated in parallel for different analyses. One portion was evaluated for sperm motility, concentration, viability, and MDA concentration, while another portion was transferred into sterile 1.5 mL microcentrifuge tubes and stored at −80 °C for up to 24 h prior to DNA extraction for microbiota analysis.

### 2.4. Assessment of Sperm Quality

Sperm motility was assessed using three distinct methods: mass movement, MOT, and PMOT. Mass movement was evaluated by placing 5 µL of fresh semen on a glass slide (without a coverslip) and observing the wave pattern of motile spermatozoa under a light microscope at 100× magnification. Activity was graded on a 0–5 scale, where 0 = no movement and 5 = vigorous waves across the field [7].

MOT and PMOT were determined using a computer-assisted sperm analysis system (IVOS version 10.0, Hamilton Thorne, Inc., Beverly, MA, USA). A 5 µL semen aliquot was loaded into a pre-warmed (25 °C) analysis chamber and examined under a phase contrast microscope equipped with a DP71 digital camera (Olympus Corporation, Tokyo, Japan). Video frames were captured at 30 frames per second (60 Hz) from three randomly selected fields. Spermatozoa with VAP < 5 µm/s were classified as non-motile, whereas those with VAP > 20 µm/s and straightness ≥ 80% were classified as progressively motile [8].

Sperm concentration was determined using a hemocytometer. A 1 μL semen aliquot was diluted with 999 μL of 4% sodium chloride solution (1:1000 dilution). The diluted sample was loaded onto the counting chamber and examined under a compound microscope at 400× magnification. Results were expressed in billions (10^9^) of sperm cells per milliliter (sperm cells/mL) [7].

Sperm viability was assessed using the eosin–nigrosin staining technique. A 5 µL semen aliquot was mixed with 10 µL of eosin–nigrosin stain, smeared onto a glass slide, air-dried, and examined under a light microscope (Olympus CH30, Olympus Corporation, Tokyo, Japan) at 1000× magnification. A total of 300 spermatozoa were evaluated per sample. Pink spermatozoa were classified as non-viable, whereas unstained cells were considered viable.

### 2.5. Determination of Lipid Peroxidation

Lipid peroxidation in rooster semen was quantified by measuring MDA concentrations using the thiobarbituric acid reactive substances assay described by Chuaychu-Noo et al. [9]. Briefly, 1,1,3,3-tetramethoxypropane (TMP; ≥99%) was used to prepare the MDA standard curve. Serial dilutions of TMP-derived MDA were reacted with 15% trichloroacetic acid (TCA) and 0.375% thiobarbituric acid (TBA) to generate the calibration curve, and absorbance was recorded at 532 nm using a UV–visible spectrophotometer (Specord 250 Plus, Analytik Jena, Jena, Germany).

For sample analysis, 250 µL of fresh semen from each rooster was diluted to 2.5 × 10^8^ sperm/mL. Each sample was mixed with 0.25 mL each of ferrous sulfate (0.2 mM) and ascorbic acid (1 mM) and incubated at 37 °C for 1 h to induce lipid peroxidation. Subsequently, 1 mL of 15% TCA and 1 mL of 0.375% TBA were added, and the reaction mixture was heated at 100 °C for 10 min to induce the formation of the pink MDA–TBA complex. After rapid cooling at 4 °C, the samples were centrifuged at 4000× *g* for 10 min, and the absorbance of the clear supernatant was measured at 532 nm. The MDA concentration was calculated from the standard curve and expressed as nanomoles per milliliter of semen.

### 2.6. DNA Extraction

Genomic DNA was extracted from 100 µL of freshly collected semen using a modified sodium dodecyl sulfate (SDS)–guanidine hydrochloride lysis method optimized for low-biomass samples [10]. Semen samples were centrifuged at 3200× *g* for 5 min, and the supernatants were removed. The cell pellets were lysed by adding 70 µL of 20% SDS, 50 µL of 7.5 M sodium acetate, 25 µL of 1 mg/mL proteinase K, and 625 µL of 5 M guanidine HCl. The mixtures were gently vortexed and incubated at 60 °C overnight. After centrifugation at 10,600× *g* for 5 min, the supernatants were transferred to clean microcentrifuge tubes. DNA was precipitated by adding 600 µL of absolute isopropanol, mixing gently, and centrifuging at 20,800× *g* for 5 min. The resulting DNA pellets were washed twice with 75% ethanol, air-dried for approximately 45 min, and resuspended in 10–30 µL of Tris–EDTA Buffer (TE buffer). Samples were incubated at 37 °C overnight to ensure complete dissolution.

DNA yield was evaluated using the Qubit™ dsDNA High Sensitivity Assay Kit (Thermo Fisher Scientific, Waltham, MA, USA) and Qubit 4 Fluorometer (Thermo Fisher Scientific, Waltham, MA, USA) and was expressed as DNA concentration (ng/µL). The mean DNA concentration was 3.56 ± 0.63 ng/µL (*n* = 30), and the total DNA yield was calculated from the elution volume. DNA purity was assessed using a NanoDrop™ 2000 spectrophotometer (Thermo Fisher Scientific, Wilmington, DE, USA), with mean A260/280 ratios of 1.83 ± 0.03. Extraction blanks (PBS only) were included as negative controls to monitor environmental and reagent contamination. These controls were processed alongside all samples and subjected to nanopore sequencing analysis. No detectable sequencing reads or microbial taxa were identified, indicating the absence of contaminant signals.

### 2.7. Quantification of Total Bacterial Load by Quantitative PCR

Total bacterial load in rooster semen was quantified by real-time quantitative PCR (qPCR) targeting the 16S rRNA gene, using universal bacterial primers (forward: 5′-CGCCAACGAGCGCAACCC-3′; reverse: 5′-CCATTGTAGCACGTGTGTAGCC-3′). These primers target conserved regions of the 16S rRNA gene and are widely used for broad-spectrum bacterial detection across diverse taxa. The expected amplicon size was approximately 130 bp. The primer set was adopted from Denman et al. [11].

Each 20 µL reaction mixture contained 10 µL of iTaq™ Universal SYBR^®^ Green Supermix (Bio-Rad Laboratories, Hercules, CA, USA), 0.5 µL of each primer (10 µM), 2 µL of template DNA (10 ng/µL), and 7 µL of nuclease-free water.

Amplification was performed on a Bio-Rad CFX96 Real-Time PCR Detection System with the following cycling conditions: initial denaturation at 95 °C for 5 min, followed by 40 cycles of 95 °C for 30 s and annealing/extension at 62 °C for 30 s. Melt-curve analysis was conducted from 65 to 95 °C with 0.2 °C increments to confirm amplification specificity. No-template controls were included in each run. Bacterial load was expressed as the relative 16S rRNA gene Cq value normalized to total DNA input (ng) for each sample.

### 2.8. Library Preparation and Nanopore Sequencing for Microbiota Profiling

High-quality genomic DNA (≥1 µg, A260/280 ≈ 1.8) was used for long-read 16S rRNA gene sequencing (ONT). A library was prepared following the manufacturer’s instructions for the Ligation Sequencing Kit (SQK-LSK114; ONT, Oxford, UK) and Native Barcoding Expansion (EXP-NBD114; version 14).

DNA repair and end-preparation were performed using the NEBNext FFPE DNA Repair Mix and NEBNext Ultra II End Repair/dA-Tailing Module (New England Biolabs, Ipswich, MA, USA). Each sample was ligated with a unique native barcode using Quick T4 DNA Ligase, incubated at room temperature for 20 min, and subsequently inactivated with EDTA. Barcoded libraries were quantified, pooled at equimolar concentrations, and purified using 0.4× AMPure XP beads (Beckman Coulter, Brea, CA, USA).

Adapter ligation was performed according to ONT guidelines, and the final library was eluted in 15 µL of elution buffer. Library concentration was determined using the Qubit dsDNA High Sensitivity Assay (Thermo Fisher Scientific, Wilmington, DE, USA). Approximately 75 µL of the sequencing mix (library, loading beads, and sequencing buffer) was loaded dropwise into the SpotON port of a FLO-MIN114 (R10.4.1) flow cell pre-primed with Flow Cell Flush and Tether solutions.

Sequencing was carried out on a MinION Mk1B device using MinKNOW software (v23.04). High-accuracy basecalling and duplex read generation were performed using Dorado (v0.5), yielding reads with a minimum quality score threshold of Q > 20. Sequencing output metrics, including read count, read length distribution, N50, and Q-scores, were extracted for quality assessment prior to downstream bioinformatics analysis.

### 2.9. Bioinformatics and Microbiota Analysis

Raw nanopore sequencing data (FAST5 format) were generated using R10.4.1 flow cells and the Native Barcoding Kit 24 V14 (SQK-NBD114.24; ONT, Oxford, UK) on the MinION Mk1C platform. Initial basecalling was performed in simplex mode using MinKNOW, followed by high-accuracy duplex re-basecalling with Dorado (ONT), as recommended for Kit 14 chemistry to achieve Q20+ read accuracy. Barcoded reads were demultiplexed using Guppy Barcoder (ONT, Oxford, UK) with the SQK-NBD114.24 configuration and stringent barcode matching parameters. Sequencing reads were obtained from all 30 semen samples and analyzed individually without pooling, allowing sample-level comparison of microbial profiles.

FASTQ reads were quality-filtered using NanoFilt with a minimum Q-score threshold of 7 and a read-length window of 1400–1700 bp to retain high-quality, full-length 16S rRNA gene sequences. Taxonomic classification and microbiota profiling were performed using the EPI2ME 16S workflow (ONT, Oxford, UK), which aligns full-length reads against the SILVA v138 reference database for species-level classification.

Bacterial composition was summarized as percentage detection frequency (%) for each detected taxon. Bacterial richness (R; total number of taxa detected) and the Berger–Parker dominance index were calculated to depict overall richness and dominance patterns. Alpha-diversity metrics, including the normalized Shannon (0–1) and Simpson (0–1) indices, were computed using the BPMSG Diversity Calculator. Detection frequencies (%) of bacterial taxa across roosters were visualized using a heatmap and descriptively summarized using IBM SPSS Statistics (version 28.0; IBM Corp., Armonk, NY, USA). These metrics were used to support descriptive comparisons among the three rooster genotypes. Additional details of the bioinformatics workflow are provided in Table S1 of the Supplementary Information.

For alpha diversity estimation, indices were calculated at the group level using aggregated taxonomic profiles. Relative abundance data from individual samples within each group were combined to generate a composite profile, from which diversity indices were derived. Consequently, a single summary value per group was obtained, and within-group variability (e.g., SD or SEM) could not be estimated.

This approach was used to provide an overall descriptive comparison of community structure among rooster types. Given the relatively small sample size per group (*n* = 10) and the focus on taxonomic profiling rather than inferential microbiome statistics, alpha diversity metrics were used for descriptive purposes only and were not subjected to statistical testing, consistent with previously reported descriptive microbiome studies (e.g., Tvrdá et al. [5]).

Sequencing depth was assessed by the total number of reads per sample after demultiplexing and quality filtering. The mean sequencing depth was 28,756 reads per sample (range: 25,860–31,566), with read counts broadly comparable across samples and no evident outliers or systematic differences among rooster types. To account for minor variation in sequencing depth, downstream analyses were performed using relative abundance (detection frequency, %). No samples were excluded due to low read counts.

### 2.10. Statistical Analysis

All statistical analyses were performed using IBM SPSS Statistics software (version 28.0). Prior to analysis, all data were screened for normality using the Shapiro–Wilk test and for homogeneity of variances using Levene’s test. When necessary, variables that violated ANOVA assumptions were log- or square-root–transformed accordingly before further statistical evaluation.

The experiment followed a CRD, with rooster type (Thai native, crossbred, and commercial) specified as the fixed factor, and individual roosters considered independent experimental units. Semen quality traits (mass movement, MOT, PMOT, viability, and sperm concentration), lipid peroxidation (MDA concentration), and total bacterial load were analyzed using one-way analysis of variance (ANOVA) under the CRD framework. When a significant main effect was detected (*p* < 0.05), Tukey’s Honestly Significant Difference test was employed for pairwise mean comparisons.

Bacterial composition data obtained from 16S rRNA gene sequencing were expressed as percentage detection frequencies (%) for each taxon and visualized as a heatmap. Since microbiota data typically do not meet the assumptions of parametric testing, the taxonomic profiles of genotypes were compared descriptively. Bacterial richness (R), the Berger–Parker dominance index, and normalized Shannon and Simpson indices (0–1) were calculated using the BPMSG Diversity Calculator.

Relationships between semen quality parameters, MDA concentrations, and bacterial load were evaluated using Pearson’s correlation analysis. Correlation strength was interpreted as slight (r < 0.20), weak (0.20–0.39), moderate (0.40–0.59), high (0.60–0.79), or very high (≥0.80). All data were expressed as mean ± standard error of the mean, and statistical significance was set at *p* < 0.05.

## 3. Results

### 3.1. Semen Quality, Oxidative Stress, and Bacterial Load

Fresh semen quality, oxidative stress indicators, and bacterial profiles are summarized in Table 1**.** Thai native roosters exhibited significantly higher semen quality than crossbred and commercial roosters. Specifically, they exhibited the highest mass motility and MOT (*p* < 0.05), with no significant differences between crossbred and commercial roosters.

PMOT, sperm concentration, and sperm viability were also significantly greater in Thai native and crossbred roosters than in commercial roosters (*p* < 0.05). Thai native roosters showed the highest sperm viability, whereas crossbred roosters exhibited intermediate values.

In contrast, commercial roosters displayed significantly higher MDA concentrations and bacterial load than the other groups (*p* < 0.05). There were no significant differences in these parameters between Thai native and crossbred roosters.

### 3.2. Bacterial Composition and Diversity

**Figure 1:**
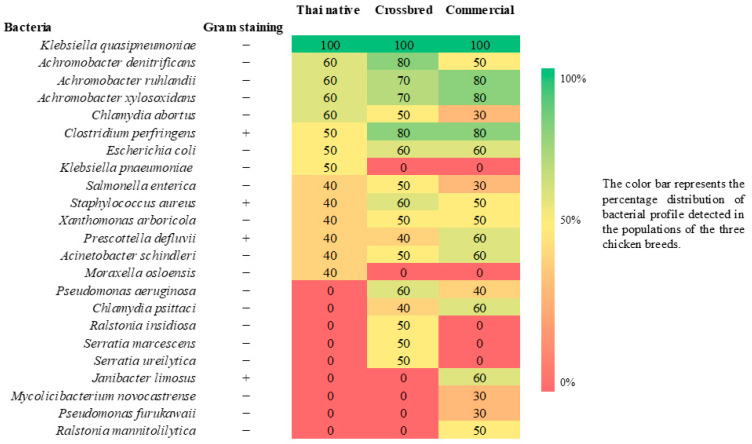
Heatmap illustrating relative detection frequency (%) of microbial taxa in semen samples from Thai native, crossbred, and commercial roosters, based on long-read 16S rRNA gene sequencing (Oxford Nanopore Technologies). The color intensity represents the prevalence of each taxon within each genotype group (green = high prevalence; red = low or undetected). The bacterial taxa are classified as Gram-positive or Gram-negative, as indicated in the second column. + and − indicate Gram-positive and Gram-negative bacteria, respectively.

Bacterial profiling revealed differences in composition and richness among the three rooster groups (Figure 1). Thai native roosters exhibited the lowest bacterial richness, with 14 detected taxa, comprising 3 Gram-positive and 11 Gram-negative bacteria.

Crossbred roosters showed a moderate bacterial profile, with 17 taxa identified (3 Gram-positive and 14 Gram-negative). In contrast, commercial roosters exhibited the highest bacterial richness, with 18 species detected: 4 Gram-positive and 14 Gram-negative.

### 3.3. Bacterial Diversity Indices

The diversity indices of the semen microbiota in Thai native, crossbred, and commercial roosters are shown in Table 2. Commercial roosters exhibited the highest species richness (18 taxa), followed by crossbred (17 taxa) and Thai native roosters (14 taxa).

In terms of community dominance, as measured by the Berger–Parker index, Thai native roosters had the highest value (0.12), whereas crossbred and commercial roosters were similar at a lower value (0.10).

The normalized Shannon index was highest in crossbred roosters (0.030), followed by commercial (0.029) and Thai native roosters (0.026). Similarly, the Simpson index was higher in Thai native roosters (0.078) compared with crossbred (0.063) and commercial roosters (0.062).

Overall, these indices indicate differences in microbial diversity and community structure among the three rooster types.

### 3.4. Correlation Analyses

Pearson’s correlation analysis (Table 3) revealed significant relationships between semen quality, oxidative status, and bacterial load.

Sperm motility traits (MOT and PMOT) positively correlated with sperm concentration and viability (*p* < 0.01). MOT and PMOT exhibited the strongest association (r = 0.749, *p* < 0.01).

Conversely, MDA levels significantly negatively correlated with all semen quality parameters (r = −0.528 to −0.712, *p* < 0.01).

Bacterial load correlated negatively with MOT (r = −0.539) and PMOT (r = −0.477) and positively with MDA levels (r = 0.482; *p* < 0.05).

## 4. Discussion

This study revealed clear differences in semen quality, oxidative status, and seminal bacterial profiles among Thai native, crossbred, and commercial roosters. Thai native roosters consistently exhibited the best semen quality, including higher sperm motility and viability, whereas commercial roosters showed comparatively poorer performance, with crossbred roosters displaying intermediate values. These findings indicate that reproductive traits and oxidative balance vary among rooster types, potentially reflecting differences in genetic background and physiological adaptation.

The observed patterns were consistent across multiple parameters, including semen quality traits, lipid peroxidation (MDA), bacterial load, and microbial community structure. This concordance suggests an integrated relationship among reproductive performance, oxidative status, and microbial characteristics, rather than indicating direct causal effects of genotype alone.

The superior semen quality of Thai native roosters aligns with previous reports highlighting enhanced resilience of indigenous breeds under tropical conditions, which may include exposure to heat stress [12,13,14]. Such resilience may be associated with long-term adaptation to tropical environments, including improved physiological stability and tolerance to oxidative stress [15,16]. In contrast, commercial broiler breeders, which have been intensively selected for growth, are more susceptible to thermal stress and oxidative imbalance, often resulting in reduced semen quality and reproductive performance [17].

In this study, commercial roosters exhibited elevated MDA levels, indicating increased lipid peroxidation and oxidative stress, which are known to impair sperm function [18,19]. The negative correlations observed between MDA and semen quality traits further support the association between oxidative stress and reduced reproductive performance.

Differences among rooster types were also observed in seminal bacterial load and composition. Commercial roosters showed higher bacterial abundance and diversity, whereas Thai native roosters exhibited lower microbial burden. These patterns suggest that variation in microbial profiles may be linked to host-related characteristics, including immune responses and environmental adaptation. Furthermore, differences in bacterial composition may contribute to variation in the oxidative environment of semen, providing a biologically plausible link between microbial presence, oxidative stress, and sperm function. However, these relationships should be interpreted as associative rather than indicative of direct genetic causation.

### 4.1. Breed Differences in Semen Quality and Lipid Peroxidation

Oxidative stress, as indicated by MDA concentrations, was closely associated with variation in semen quality among rooster types. Commercial roosters showed higher oxidative stress and poorer semen quality, whereas Thai native roosters exhibited lower oxidative stress and better semen traits, with crossbred roosters displaying intermediate patterns.

Spermatozoa are highly susceptible to oxidative damage due to their elevated polyunsaturated fatty acid content. Lipid peroxidation disrupts membrane integrity, impairs mitochondrial function, and reduces ATP availability, ultimately compromising sperm motility and fertilization potential [20]. Consistent with these mechanisms, strong negative correlations were observed between MDA and MOT (r = −0.712), PMOT (r = −0.700), and viability (r = −0.562) (Table 3), supporting the association between oxidative stress and impaired sperm function.

These findings align with previous reports showing that heat stress and metabolic load increase ROS production, leading to lipid peroxidation and reduced sperm quality in poultry [7,21,22]. The observed oxidative patterns among rooster types may reflect differences in antioxidant capacity and physiological adaptation to environmental stressors.

Indigenous and slow-growing breeds, such as Thai native chickens, have been reported to exhibit more effective antioxidant defenses under tropical conditions, which may contribute to their lower oxidative stress and improved semen quality. Conversely, commercial broiler breeders, selected for rapid growth, are more prone to oxidative imbalance due to higher metabolic demands and reduced resilience [23]. These observations are consistent with previous reports linking differences among rooster types with variation in lipid peroxidation and seminal microbial characteristics [6].

### 4.2. Bacterial Composition and Patterns Among Rooster Types

Distinct patterns of seminal bacterial composition were observed among rooster types. Commercial roosters exhibited greater bacterial diversity and a more evenly distributed microbial profile, whereas Thai native roosters showed lower diversity and reduced taxonomic complexity. Crossbred roosters displayed intermediate patterns.

These differences suggest variation in microbial communities associated with rooster types, consistent with previous reports describing associations between host background and reproductive tract microbiota [6,24]. The observed patterns may reflect differences in environmental microbial exposure, housing conditions, and host-related factors such as mucosal immune regulation [24,25].

However, diversity metrics alone do not fully explain the functional relevance of these microbial profiles. Therefore, the potential roles of specific bacterial taxa in relation to oxidative status and sperm functional traits are considered in the following section.

### 4.3. Ecological Grouping of Seminal Bacteria as an Interpretative Framework

To better interpret the functional implications of seminal bacterial profiles, detected bacterial taxa were categorized into four ecological groups: (i) core commensals, (ii) environmental taxa, (iii) classical pathogens, and (iv) opportunistic Gram-negative bacteria (Table S2). This framework enables a structured discussion of how bacterial groups relate to semen quality and oxidative patterns across rooster types within an associative context.

Core commensals, including *Achromobacter* spp. and *Klebsiella quasipneumoniae*, were consistently detected across all groups and are generally considered non-pathogenic residents of the male reproductive tract [24,26], and probably maintain mucosal homeostasis and play protective roles against colonization by harmful taxa [27]. Their presence was not associated with impaired semen quality, suggesting a neutral or potentially stabilizing role in maintaining microbial balance.

Environmental bacteria, such as *Ralstonia* and *Moraxella* spp., were detected sporadically, primarily in crossbred and commercial roosters. Their intermittent occurrence likely reflects transient environmental exposure associated with housing or handling conditions, rather than stable colonization [28,29], indicating possible differences in microbial exposure or mucosal filtering efficiency among rooster types.

In contrast, classical pathogens, including *Salmonella enterica* and *Staphylococcus aureus*, were more frequently detected in commercial and crossbred roosters. These organisms are known to impair sperm function through inflammatory or direct membrane-damaging mechanisms [1,30], and their higher detection frequency coincided with reduced semen quality.

Opportunistic Gram-negative bacteria, such as *Escherichia coli*, *Pseudomonas aeruginosa*, and *Enterobacter cloacae*, were most prevalent in commercial roosters. These taxa are associated with endotoxin production and cellular stress responses [1,31], which may contribute to altered semen quality, consistent with the oxidative patterns observed in this study.

Overall, these findings suggest that bacterial identity and ecological role, rather than bacterial presence alone, are relevant to variation in semen quality among rooster types.

### 4.4. Relationship Between Seminal Microbiota, Oxidative Stress, and Semen Traits

Building upon the ecological grouping outlined in Table S2, opportunistic Gram-negative bacteria were the taxa most consistently associated with reduced semen quality. These bacteria were detected more frequently in crossbred and commercial roosters than in Thai native roosters, paralleling trends in bacterial load and oxidative status.

The outer membranes of Gram-negative taxa contain LPS, which can activate inflammatory signaling pathways and promote the production of reactive oxygen species, leading to cellular stress and sperm dysfunction [32,33]. In this study, higher detection frequencies of these taxa were associated with poorer semen quality and increased oxidative stress, as reflected by the MDA results described earlier.

Notably, oxidative parameters appeared to be more closely related to bacterial composition than to overall bacterial abundance, suggesting that functional characteristics of specific taxa may shape the oxidative environment of semen.

Host defense mechanisms, including antioxidant systems and immune regulation, likely contribute to these interactions. Thai native roosters maintained superior semen quality despite the presence of some bacterial taxa, which may reflect lower exposure to high-risk Gram-negative bacteria and greater physiological resilience. These findings support an integrative and associative framework in which microbial composition, oxidative balance, and semen traits are interconnected and vary among rooster types, without implying direct genetic causation.

It is important to emphasize that the present study represents a comparative evaluation among rooster types that differ not only in genetic background but also in broader physiological characteristics. Although birds were maintained under standardized housing conditions, intrinsic differences—including metabolic rate, reproductive physiology, and historical selection pressures—may contribute to the observed variation.

In this context, the findings support an integrative and associative framework in which seminal microbial composition, oxidative balance, and semen quality are interrelated, without implying direct genetic causation. Future studies using controlled breeding designs or genomic approaches are needed to further disentangle these effects.

## 5. Conclusions

Thai native roosters exhibited superior semen quality compared to crossbred and commercial roosters, as reflected by higher sperm motility and viability and lower lipid peroxidation. Crossbred roosters showed intermediate values, whereas commercial roosters displayed comparatively lower semen quality, highlighting differences among rooster types in reproductive performance and oxidative status.

Seminal bacterial profiling revealed clear differences among rooster types in bacterial load and taxonomic patterns. Thai native roosters exhibited a lower bacterial burden and reduced taxonomic complexity, whereas commercial roosters showed higher detection frequencies of opportunistic Gram-negative bacteria. These microbial patterns were associated with increased oxidative stress and reduced semen quality.

The findings support an integrative and associative framework in which semen quality reflects interactions among rooster type, microbial composition, and oxidative balance under typical production conditions. Strategies aimed at reducing bacterial burden and oxidative stress may improve semen preservation and fertility outcomes. Further research is needed to evaluate targeted interventions, including antioxidant supplementation and antimicrobial substances, within microbiome-informed reproductive management systems.

## Figures and Tables

**Table 1:** Fresh semen quality parameters, lipid peroxidation (malondialdehyde, MDA) levels, bacterial load (Cq value), and number of bacterial taxa identified in Thai native, crossbred, and commercial roosters (mean ± SEM; *n* = 10 per group).

Parameters	Thai Native	Crossbred	Commercial
**A. Semen Quality**			
Mass movement (score 1–5)	4.48 ± 0.02 ^a^	4.40 ± 0.04 ^b^	4.38 ± 0.03 ^b^
Total motility (MOT, %)	91.24 ± 1.36 ^a^	89.56 ± 1.31 ^b^	88.93 ± 1.91 ^b^
Progressive motility (PMOT, %)	80.46 ± 2.38 ^a^	79.29 ± 2.24 ^a^	75.96 ± 2.72 ^b^
Sperm concentration (×10^9^/mL)	5.05 ± 0.80 ^a^	4.94 ± 0.51 ^a^	4.52 ± 0.41 ^b^
Sperm viability (%)	94.21 ± 3.11 ^a^	91.83 ± 2.21 ^ab^	90.24 ± 2.63 ^b^
**B. Oxidative and Microbial Load**			
MDA (µmol/mL)	1.04 ± 0.04 ^a^	1.18 ± 0.02 ^a^	1.25 ± 0.06 ^b^
Bacterial load (Cq value) ^1^	23.12 ± 0.40 ^a^	22.23 ± 0.76 ^a^	20.29 ± 0.61 ^b^
**C. Bacterial Species Detected**			
Total bacteria detected	14 species	17 species	18 species
– Gram-negative	11	14	14
– Gram-positive	3	3	4

^1^ Bacterial load was expressed as quantification cycle (Cq) values based on qPCR; lower Cq values indicate higher bacterial abundance (greater initial template), whereas higher Cq values indicate lower bacterial load. Values within a row with different superscript letters (^a^, ^b^) are significantly different (*p* < 0.05).

**Table 2:** Descriptive bacterial diversity indices of semen microbiota in Thai native, crossbred, and commercial roosters.

Diversity Index	Thai Native	Crossbred	Commercial
Species Richness	14	17	18
Community Dominance (Berger–Parker Index)	0.12	0.10	0.10
Alpha Diversity (normalized)			
Shannon index (0–1)	0.026	0.030	0.029
Simpson index (0–1)	0.078	0.063	0.062

Richness represents the total number of detected microbial species. The Berger–Parker index reflects the proportional dominance of the most abundant taxon, where lower values indicate reduced dominance. Normalized Shannon and Simpson indices (scaled 0–1) describe alpha diversity and community evenness. Alpha diversity indices were calculated at the group level from aggregated taxonomic profiles and are presented for descriptive purposes only; therefore, measures of within-group variability (e.g., SD or SEM) are unavailable.

**Table 3:** Pearson’s correlation coefficients (r) among semen quality parameters, lipid peroxidation (MDA), and bacterial load in rooster semen.

Parameters	Concentration	MOT	PMOT	Viability	MDA	Bacteria Load
**Mass movement**	0.584 **	0.458 *	0.437 *	0.243	−0.683 **	−0.261
**Concentration**	1	0.574 **	0.463 *	0.466 **	−0.528 **	−0.243
**MOT**		1	0.749 **	0.592 **	−0.712 **	−0.539 **
**PMOT**			1	0.493 **	−0.700 **	−0.477 **
**Viability**				1	−0.562 **	−0.460 *
**MDA**					1	0.482 *

Abbreviations: MOT = total motility; PMOT = progressive motility; MDA = malondialdehyde. *p* < 0.05 = significant (*), *p* < 0.01 = highly significant (**).

## Data Availability

The data are available upon request from the corresponding author.

## Supplementary Materials

The following supporting information can be downloaded at: https://www.mdpi.com/article/10.3390/ani16091380/s1, Table S1: Bioinformatic workflow verification; Table S2. Ecological grouping and prevalence (%) of bacterial taxa detected in rooster semen.
