# Supplementary material for: Comparative Variation and Associations Among Seminal Microbiota, Oxidative Status, and Semen Quality in Different Rooster Types
Source: Animals (Basel). 2026 Apr 30;16(9):1380. doi: 10.3390/ani16091380 (PMC13162573; doi:10.3390/ani16091380)
Supplement: Supplementary file 1 [file animals-16-01380-s001.zip › Table S1.pdf]

Table S1. Bioinformatic workflow verification

Item	Verification
Bioinformatic tools used	EPI2ME 16S workflow (taxonomy), BPMSG Diversity Calculator (alpha diversity), SPSS v28 (descriptive statistics).
Reference database	SILVA v138 for full-length 16S rRNA classification.
Quality filtering	NanoFilt ($Q \geq 7$; read length 1,400–1,700 bp).
Statistical summary	SPSS v28 for percentage occurrence analysis.
Diversity indices	Shannon, Simpson (BPMSG Diversity Calculator).#
