# Supplementary material for: Comparative Variation and Associations Among Seminal Microbiota, Oxidative Status, and Semen Quality in Different Rooster Types
Source: Animals (Basel). 2026 Apr 30;16(9):1380. doi: 10.3390/ani16091380 (PMC13162573; doi:10.3390/ani16091380)
Supplement: Supplementary file 1 [file animals-16-01380-s001.zip › Table S2.pdf]

Table S2. Ecological grouping and prevalence (%) of bacterial taxa detected in rooster semen

Ecological group	Bacterial species	Thai native (%)	Crossbred (%)	Commercial (%)
**Core commensals**	*Klebsiella quasipneumoniae*	100	100	100
	Achromobacter spp.	40	50	70
**Opportunistic Gram-negative**	*Pseudomonas aeruginosa*	0	60	40
	Pseudomonas furukawaii	20	40	60
	Escherichia coli	40	50	60
	Serratia marcescens	0	50	30
	Ralstonia insidiosa	10	20	30
	Acinetobacter schindleri	30	40	50
**Environmental / water-associated**	*Ralstonia mannitolilytica*	20	30	40
	Achromobacter xylosoxidans	30	40	50
	Moraxella spp.	10	20	30
	Prescottella equi	20	30	40
	Mycolicibacterium novocastrense	10	20	30
**Classical pathogens (non-reproductive origin)**	*Salmonella enterica*	40	50	30
	Clostridium perfringens	20	30	40
	Staphylococcus aureus	30	40	50
	Chlamydia abortus	20	30	30
	Chlamydia psittaci	0	20	20

Bacterial taxa were classified into ecological groups based on ecological niche, pathogenic potential, and prevalence patterns. Prevalence indicates the percentage of samples in which each taxon was detected within each genotype.
